# Emergence of Amoxicillin-Resistant Variants of Spain^9V^-ST156 Pneumococci Expressing Serotype 11A Correlates with Their Ability to Evade the Host Immune Response

**DOI:** 10.1371/journal.pone.0137565

**Published:** 2015-09-14

**Authors:** Leire Aguinagalde, Bruno Corsini, Arnau Domenech, Mirian Domenech, Jordi Cámara, Carmen Ardanuy, Ernesto García, Josefina Liñares, Asunción Fenoll, Jose Yuste

**Affiliations:** 1 Spanish Pneumococcal Reference Laboratory, Centro Nacional de Microbiología, Instituto de Salud Carlos III, Madrid, Spain; 2 Microbiology Department, Hospital Universitari de Bellvitge-IDIBELL-Barcelona University, Barcelona, Spain; 3 CIBER de Enfermedades Respiratorias (CIBERES), Madrid, Spain; 4 Centro de Investigaciones Biológicas-CSIC, Madrid, Spain; Centers for Disease Control & Prevention, UNITED STATES

## Abstract

Capsular switching allows pre-existing clones of *Streptococcus pneumoniae* expressing vaccine serotypes to escape the vaccine-induced immunity by acquisition of capsular genes from pneumococci of a non-vaccine serotype. Here, we have analysed the clonal composition of 492 clinical isolates of serotype 11A causing invasive disease in Spain (2000–2012), and their ability to evade the host immune response. Antibiograms, serotyping and molecular typing were performed. The restriction profiles of *pbp2x*, *pbp1a* and *pbp2b* genes were also analysed. Interaction with the complement components C1q, C3b, C4BP, and factor H was explored whereas opsonophagocytosis assays were performed using a human cell line differentiated to neutrophils. Biofilm formation and the polymorphisms of the major autolysin LytA were evaluated. The main genotypes of the 11A pneumococci were: ST62 (447 isolates, 90.6%), followed by ST6521 (35 isolates, 7.3%) and ST838 (10 isolates, 2.1%). Beta lactam resistant serotype 11A variants of genotypes ST838 and ST6521 closely related to the Spain^9V^-ST156 clone were first detected in 2005. A different pattern of evasion of complement immunity and phagocytosis was observed between genotypes. The emergence of one vaccine escape variant of Spain^9V^-ST156 (ST6521^11A^), showing a high potential to avoid the host immune response, was observed. In addition, isolates of ST6521^11A^ showed higher ability to produce biofilms than ST838^11A^ or ST62^11A^, which may have contributed to the emergence of this PEN-resistant ST6521^11A^ genotype in the last few years. The emergence of penicillin-resistant 11A invasive variants of the highly successful ST156 clonal complex merits close monitoring.

## Introduction

*Streptococcus pneumoniae* is the most common etiologic agent of acute otitis media, community-acquired pneumonia, non-epidemic meningitis and a major cause of bacterial sepsis, especially in young children and older adults [1,2]. The capsule is considered the main pneumococcal virulence factor and, up to 96 distinct capsular polysaccharides (CPS) have been described [3]. Pneumococci of various serotypes, such as 1 and 7F, show low genetic diversity, whereas serotype 19A CPS is expressed by several genotypes that show geographical and temporal variations [4–6]. Exchange of capsular synthetic loci between pneumococcal strains (capsular switching) provides a mechanism of immune-escape to PCV7 serotype clones [7–9].

Invasive pneumococcal disease (IPD) is a complex process in which several factors are involved, including the virulence of the infective strain and the host immune response. Recognition and clearance by the complement system and phagocytic cells is critical to prevent the development of IPD [10,11]. Complement activation leads to the formation of the key complement component C3b that plays a major role in innate and adaptive immunity to pneumococcus [12]. Among the three complement cascades, the classical pathway (CP) has been shown to be essential against *S*. *pneumoniae* [13,14]. Establishment of IPD is preceded by nasopharyngeal colonization where the bacterium is located as a sessile microbial community avoiding complement immunity and phagocytosis by inducing biofilm formation [15]. The reasons underlying the different colonizing capacities observed between pneumococcal strains are unclear, although the chemical composition and structure of the CPS may play roles [16]. An additional threat for the outcome of the infection, is the emergence of clinical isolates with high levels of antibiotic resistance [4,6,7,17–20]. In pneumococci, resistance to β-lactam antibiotics is due to modified penicillin-binding proteins (PBPs), mainly PBP1A, PBP2B and PBP2X, which reduce the affinity for these antibiotics [21]. Both *pbp1a* and *pbp2x* genes are located flanking the capsular locus, and in some cases, the recombination fragment also includes partial or intact *pbp1a* and *pbp2x* genes, leading to the acquisition of a new CPS together with new PBP genes [20].

Over the last three decades, invasive serotype 11A pneumococci received at the Spanish Pneumococcal Reference Laboratory (SPRL) were usually penicillin-susceptible. However, since 2005, emergence of penicillin-resistant serotype 11A pneumococci has been identified resulting in a significant concern as this serotype is not included in the current conjugate vaccines. Our study shows that certain genotypes within serotype 11A, might have an evolutionary advantage to persist and spread in the future because they more efficiently avoid the host immune response, which may explain the emergence of this serotype in recent years.

## Materials and Methods

### Ethics statement

Healthy subjects gave their written informed consent prior to the collection of their serum. The Instituto de Salud Carlos III Ethic Committee approved this study (Approval Reference: PA 52_2011-v2).

### Bacterial isolates, susceptibility testing and growth conditions

The study included all pneumococcal invasive isolates (n = 26124) received at the SPRL during the period 2000–2012 from 190 Spanish hospitals located all over the country. Susceptibility to penicillin (PEN), amoxicillin (AMX) and cefotaxime/ceftriaxone (CTX/CRO) was determined as previously described [22]. *S*. *pneumoniae* strains selected for immunological studies were cultured on blood agar plates at 37°C in a CO_2_ atmosphere, or in Todd-Hewitt broth supplemented with 0.5% yeast extract, to an optical density at 550 nm (OD_550_) of 0.5. Stocks were stored at -70°C in 10% glycerol as single use aliquots for further experiments.

### Serotyping and molecular typing

Isolates were serotyped by the Quellung reaction, dot blot assay [23] and/or real-time PCR [24] whereas molecular characterization was performed by PFGE and MLST [20,25]. Since serotype 11A in Spain has been associated with the fully β-lactam-susceptible clone ST62, we performed a randomized selection of 70 susceptible isolates using PFGE/MLST analysis, in order to confirm their genotype. In addition, all serotype 11A pneumococci with a decreased susceptibility to β-lactams (n = 45), were genotyped by PFGE. Since all PEN-resistant pneumococci shared an identical PFGE pattern, five selected isolates were subjected to MLST. The remaining PEN-resistant isolates were chosen for *aroE* and *ddl* sequencing, since ST838 and ST6521 each differ from ST156 within one or both of these MLST targets.

### PBPs fingerprinting

The restriction fragment length polymorphism (RFLP) profiles of *pbp1A*, *pbp2X* and *pbp2B* of β-lactam-resistant 11A pneumococci were analysed as previously described [20]. The PBP gene RFLP patterns from the β-lactam-resistant 11A pneumococcal isolates were visually compared with those of the control strains ATCC 700671 (Spain^9V^-ST156) and clinical isolate HUB10926 (11A/ST62). In addition, at least two isolates belonging to each ST were selected for PBP-sequencing [20].

### Complement factors binding to *S*. *pneumoniae*

Fifteen invasive clinical isolates of serotype 11A (five of each ST62, ST838 and ST6521) were selected to evaluate their interaction with complement factors. C1q, C3b, FH and C4BP deposition were analyzed using a flow cytometry assay as previously described [14,15,26]. The results were expressed as a relative % fluorescence index (RFI) that measures not only the proportion of fluorescent bacteria positive for the host serum component investigated but also the intensity of fluorescence that quantify the amount bound [26,27].

### Opsonophagocytosis assays

Experiments investigating phagocytosis were performed using a flow cytometry assay including *S*. *pneumoniae* labeled with 5, 6-carboxyfluorescein succinimidyl ester (FAM-SE; Molecular Probes) and human HL-60 cells (CCL-240; ATCC) differentiated to granulocytes [14,15,27,28]. Infection assays were performed with a ratio of 10 bacteria per cell. Results were expressed as a RFI defined as the proportion of positive cells for fluorescent bacteria multiplied by the geometric mean of fluorescence intensity, which correlates with the amount of bacteria phagocytosed per cell [26,27].

### Biofilm quantification

Biofilm formation was determined as the ability of pneumococcal cells to adhere to the walls and base of 96-well polystyrene microtiter plates (Costar 3595; Corning) as previously described [15,29]. After 6 h of incubation at 34°C, the biofilm formed was stained with 0.2% crystal violet and rinsed to remove non-adherent bacteria. After solubilizing the biofilm in 95% ethanol (200 μl per well), OD_595_ was determined using a microplate reader (Anthos 2020).

### Confocal microscopy

HL-60 cells were infected with FAM-SE labeled pneumococcal strains as described before [26]. DNA was stained with Hoechst (Invitrogen) whereas actin cytoskeleton was stained with rhodamine-phalloidin (Invitrogen) [26]. Phagocytosis was analyzed with a Leica spectral SP5 confocal microscope using the Leica software (LAS-AF). Biofilm formation was observed using a Leica TCS-SP2-AOBS-UV confocal microscope. Briefly, pneumococcal isolates were grown on glass-bottom dishes (WillCo-dish, WillCo Wells) for 6 h at 34°C and stained with the *Bac*Light kit showing living (green fluorescence) and dead (red fluorescence) bacteria, as previously described [29].

### Statistical analysis

Data are representative of results obtained from at least three repeated independent experiments, and each data point represents the mean and standard deviations (SD) for 3 to 5 replicates. Statistical analysis was performed by using two-tailed Student’s t test (for two groups), whereas analysis of variance (ANOVA) followed by a Dunnett post hoc test were chosen for multiple comparisons. GraphPad InStat version 5.0 (GraphPad Software, San Diego, CA) was used for statistical analysis. Differences were considered statistically significant with *P* < 0.05 (*) and highly significant with *P* < 0.01 (**) or *P* < 0.001 (***).

## Results

### Trends in epidemiology of pneumococcal serotype 11A

**Fig 1:**
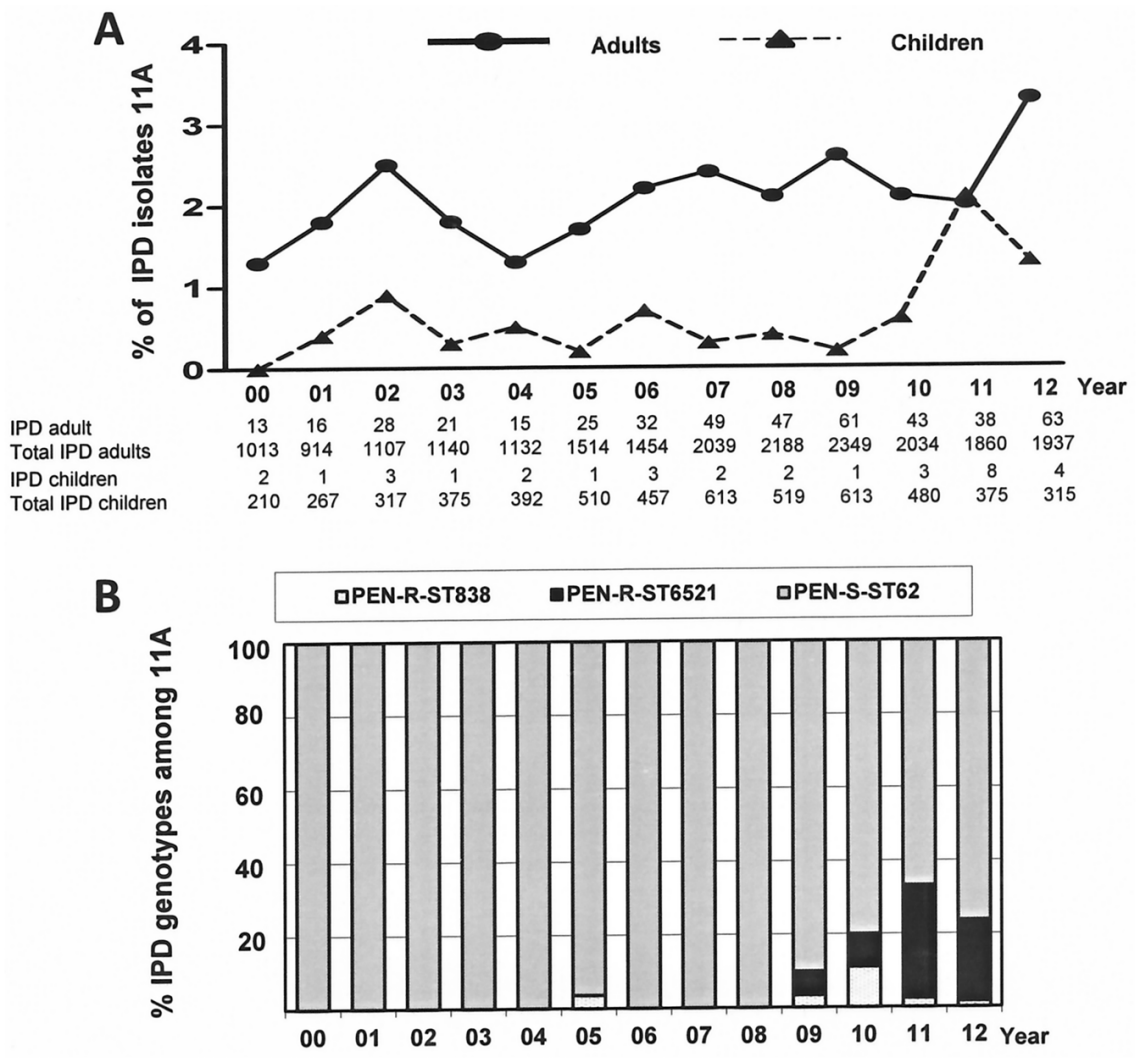
Epidemiology of serotype 11A pneumococci in Spain. (A) Invasive serotype 11A isolate recovery over time from children and adults in Spain (2000–2012). (B) Distribution of serotype 11A genotypes over the study period. PEN-R (penicillin-resistant MIC ≥2μg/ml), PEN-S (penicillin susceptible MIC ≤0.06 μg/ml). All PEN-R isolates were genotyped whereas only 70 PEN-S serotype 11A isolates were subjected to MLST analysis.

A total of 492 invasive isolates (1.9%) of 26124 invasive pneumococci sent to the SPRL from 2000 to 2012 expressed serotype 11A. Of them, 451 were isolated from adult patients (1.7% of overall adult IPD) and 31 isolates were obtained from children (0.1% of children IPD). Fig 1a shows the trends in frequency of serotype 11A among invasive pneumococci from children and adults in Spain. Serotype 11A was isolated more frequently among adults (*P* <0.001). Overall, the proportion of serotype 11A among invasive pneumococci increased from 1.3% in 2000 (13 isolates) to 3.3% in 2012 (67 isolates) (*P* <0.01). In the last three years, coinciding with the introduction of PCV-13 in Spain, the increase of serotype 11A was also significant from 2.1% in 2010 (46 isolates) to 3.3% in 2012 (67 isolates) (*P* <0.05) (Fig 1A). Although our data shows an increase in the proportion of serotype 11A IPD, this increase was modest, with a peak of 3.3% of total IPD in 2012. All invasive isolates of serotype 11A collected from 2000 to 2004, were susceptible to PEN (MIC ≤0.06 μg/ml), CTX/CRO (MIC ≤0.06 μg/ml) and AMX (MIC ≤0.06 μg/ml). In 2005 highly penicillin-resistant (MIC > 2 μg/ml) serotype 11A isolates were detected (Fig 1B). These isolates markedly increased during 2010–2012, reaching a proportion of 23.8% of 11A IPD isolates recovered during 2012 (15 PEN-resistant isolates vs 48 PEN-susceptible isolates) (Fig 1B) (*P* <0.001).

#### Molecular characterization

All PEN-susceptible serotype 11A pneumococci belonged to the same PFGE cluster and were related to ST62. The molecular characterization of PEN-resistant serotype 11A isolates demonstrated the existence of two genotypes: ST6521^11A^ (n = 35; 7.3%) and ST838^11A^ (n = 10; 2.1%). ST838^11A^ was first detected in 2005 but with its peak during 2009 and 2010 (Fig 1B). In contrast, ST6521^11A^ was detected during 2009, increasing to 31% in 2011 (Fig 1B). Currently ST6521^11A^ is the predominant PEN-resistant clone in children and adults (Fig 1B). Both ST838^11A^ and ST6521^11A^ are closely related to ST156, which originated in the highly successful Spain^9V^-ST156 clone (Table 1). ST6521^11A^ is a single locus variant (SLV) of ST838 and a double locus variant (DLV) of ST156; ST838^11A^ is a SLV of ST156 (Table 1). In terms of penicillin resistance, we have seen an increase of PEN-resistant IPD isolates of serotype 11A from 1 isolate in 2008 (0.7% of total PEN-R IPD isolates) to 14 isolates in 2012 (10% of total PEN-R IPD isolates). The number of PEN-R 11A during 2012 was only exceeded by serotype 14 (35%) and serotype 19A (38.3%).

**Table 1:** Characteristics of Major Genotypes of Serotype 11A and Spain^9V^-ST156 Clone.

								β-lactam MIC (μg/ml)^b^					
Sequence Type	MLST alleles^a^							PEN	CTX	AMX	PBP1A/2B/2X allele^c^		
**ST62** ^**d**^	2	5	29	12	16	3	14	≤0.06	≤0.06	≤0.5	A	A	A
**ST156** ^**e**^	7	11	10	1	6	8	1	2	1	2	B	B	B
**ST838** ^**f**^	7	11	10	1	6	8	90	2–4	1–2	4–16	B	C	B
**ST6521** ^**f**^	8	11	10	1	6	8	90	1–4	0.5–2	4–16	B	C	B

^a^ *aroE*, *gdh*, *gki*, *spi*, *recP*, *xpt*, *ddl* alleles.

^b^PEN, penicillin (susceptible: MIC ≤0.06 μg/ml, CLSI meningitis criteria); CTX-CRO, cefotaxime-ceftriaxone (susceptible: MIC ≤1 μg/ml, CLSI non-meningitis criteria); AMOX, amoxicillin (susceptible: MIC ≤2 μg/ml, CLSI non-meningitis criteria).

^c^Capital letters were used to define different alleles of each PBP.

^d^Only 70 Pen-S isolates were typed.

^e^ATCC 700671 used as representative isolate of this genotype. No ST156 pneumococci expressing serotype11A were detected in the present study.

^f^All Pen-R isolates were typed.

#### PBP fingerprinting

All ST 838 and ST6521 isolates shared identical PCR-RFLP profiles and DNA sequences for *pbp1a*, *pbp2b* and *pbp2x*, associated with similar β-lactam MICs (PEN, 2–4 μg/ml; CTX 1–2μg/ml; AMX 4–8 μg/ml). The two PEN-R 11A strains shared the same *pbp1a* and *pbp2x* RFLP profiles with ATCC 700671 (9V-ST156). These three resistant strains shared identical *pbp1a* sequences and closely related *pbp2x* (5 amino acid substitutions not predicted to affect resistance). There were 21 residues that differed between the *pbp2b* of the two PEN-R 11A strains compared to ATCC 700671, again at positions not predicted to be associated with ß-lactam resistance.

### Differential recognition of 11A clones by the complement system

**Fig 2:**
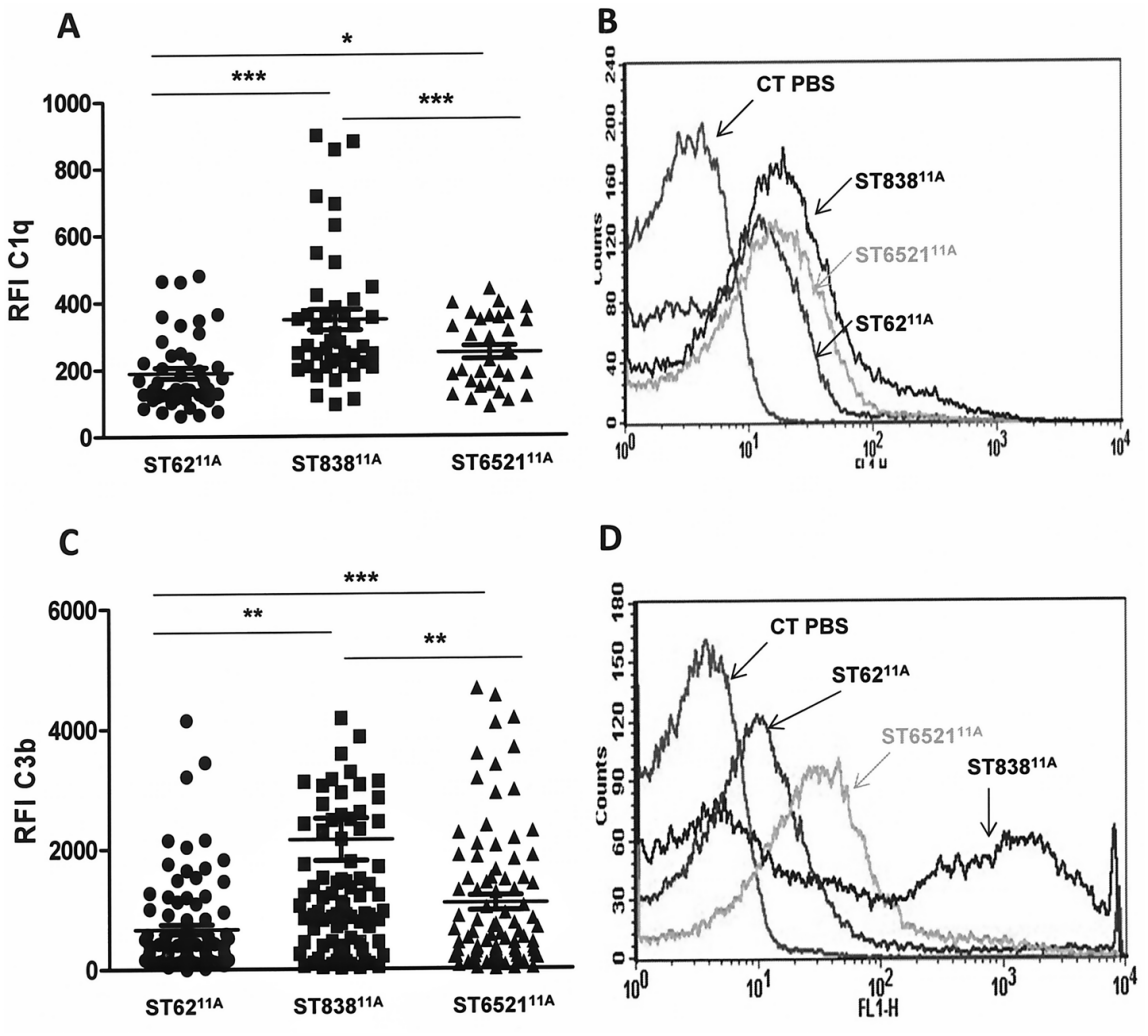
Recognition of the different genotypes within serotype 11A by the complement system. (A) Deposition of the first component C1q of the classical pathway on the surface of the different isolates. (B) Example of a flow cytometry histogram for C1q deposition. (C) C3b deposition on the surface of strains of the different genotypes. (D) Example of a flow cytometry histogram for C3b deposition. Error bars represent the standard deviations (SDs) and asterisks indicate statistical significance between the different genotypes. For the comparison between isolates of genotypes ST62^11A^ and ST6521^11A^ compared to ST838^11A^, *P* < 0.001 (one-way ANOVA with Dunnett’s post *hoc* test).

The successful emergence of certain pneumococcal clones could be linked to increased efficiency in avoiding the host immune system. The more successful emergence of ST6521^11A^ relative to ST838^11A^ might be in part due to avoidance of C1q and C3b deposition (Fig 2). Although less effective in this regard than the prevalent ST62^11A^, within the ST6521^11A^ genetic background these results potentially reflect an evolutionary advantage in escaping the complement system (Fig 2).

**Fig 3:**
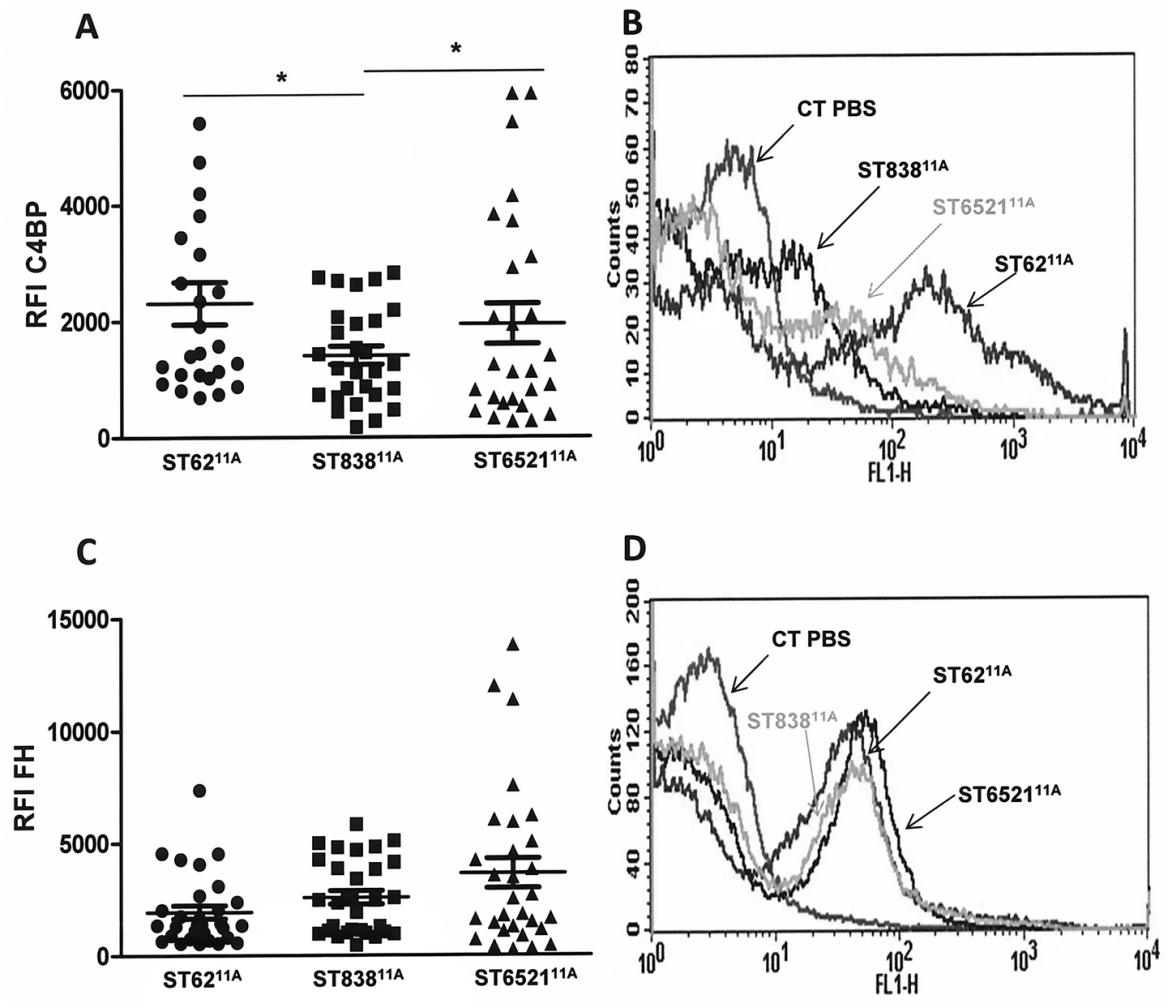
Recruitment of complement down-regulators C4BP and factor H by the different genotypes of serotype 11A. (A) C4BP binding to the surface of the different isolates. (B) Example of a flow cytometry histogram for C4BP deposition. (C) Recruitment of factor H on the surface of the different genotypes. (D) Example of a flow cytometry histogram for factor H deposition. Error bars represent the standard deviations (SDs) and asterisks indicate statistical significance between the different genotypes.

Recruitment of fluid phase down-regulators such as C4BP and FH by microbial pathogens reduces the activation of the CP or alternative pathway respectively [30]. Binding to C4BP but not to FH was increased in isolates belonging to ST62^11A^ and ST6521^11A^ compared to ST838^11A^, potentially associating the reduced opsonization by C1q and C3b with enhanced C4BP recruitment (Fig 3).

### Opsonophagocytosis of serotype 11A

**Fig 4:**
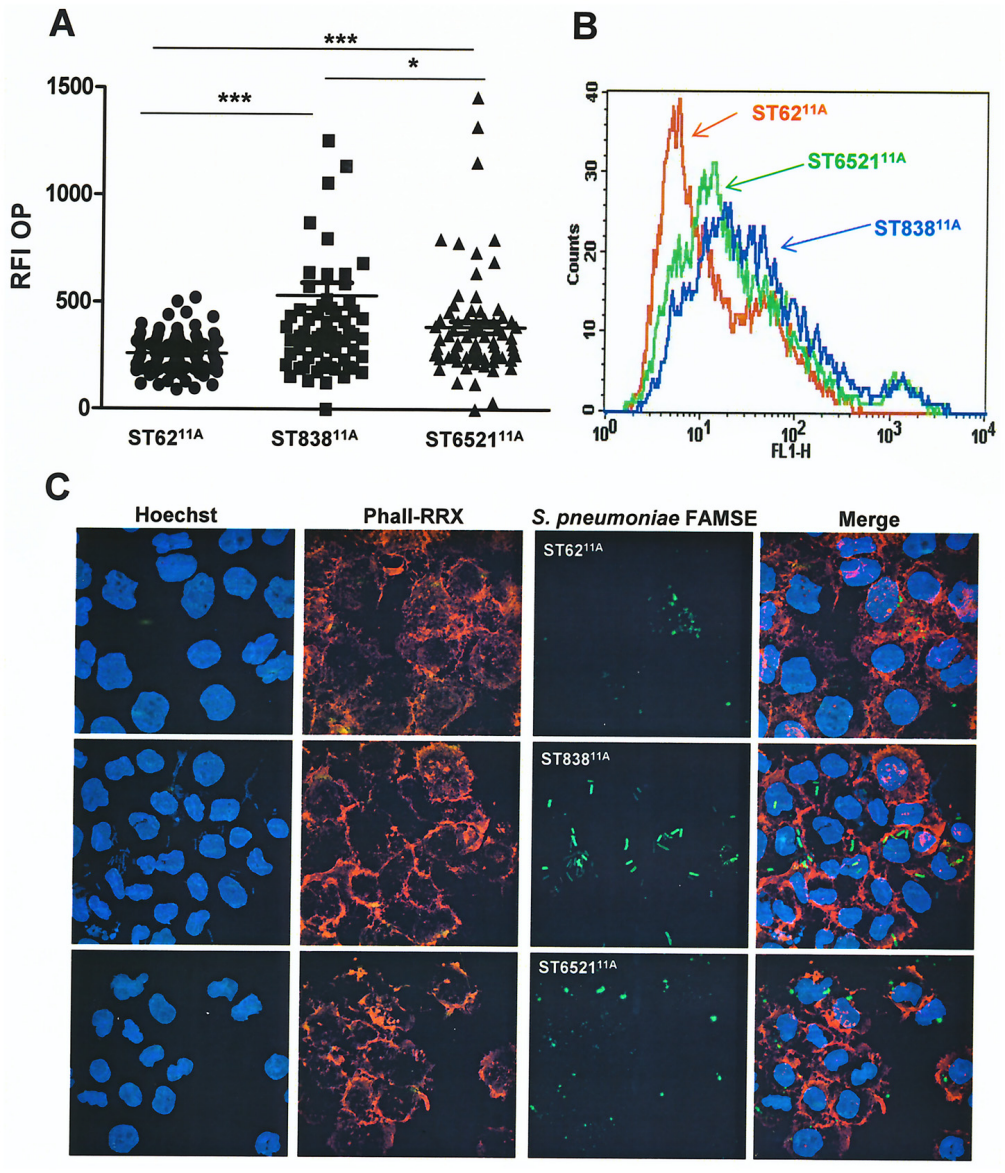
Opsonophagocytosis by HL-60 cells differentiated to human neutrophils. (A) Opsonophagocytosis of the different genotypes within serotype 11A by flow cytometry. (B) Example of a flow cytometry histogram for phagocytosis. (C) Opsonophagocytosis of the different isolates fluorescently labeled with FAM-SE detected by confocal microscopy. DNA was stained by Hoechst, actin cytoskeleton was visualized with Rhodamine-Phalloidin (RRX) staining and bacterial isolates of the different genotypes were fluorescently labeled with FAM-SE. Error bars represent the standard deviations (SDs) and asterisks indicate statistical significance between the different genotypes. For the comparison between isolates of genotypes ST62^11A^ and ST6521^11A^ compared to ST838^11A^, *p* < 0.001 (one-way ANOVA with Dunnett’s post hoc test).

To investigate the interaction of the three different genotypes with human neutrophils, an opsonophagocytosis assay was performed. Our findings indicated that invasive clinical isolates of ST62^11A^ are more resistant to phagocytosis than those of ST6521^11A^ and ST838^11A^ (Fig 4). Among PEN-resistant genotypes, isolates of ST6521^11A^ have a higher ability to divert pneumococcal phagocytosis than isolates of ST838^11A^ (Fig 4).

### *In vitro* biofilm formation by serotype 11A IPD isolates

**Fig 5:**
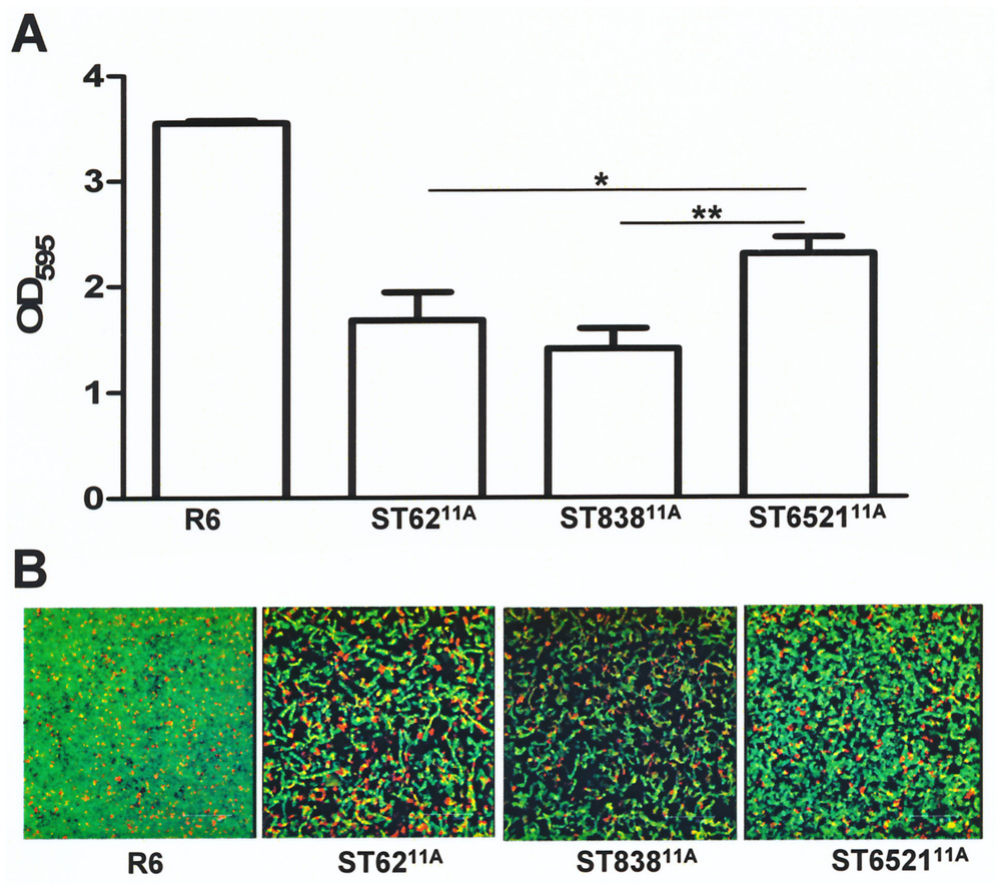
Biofilm forming capacity of serotype 11A pneumococcal isolates. (A) Biofilm formation of *S*. *pneumoniae* R6 strain and isolates of genotypes ST62^11A^, ST838^11A^ and ST6521^11A^. (B) Confocal microscopy showing images of the biofilms formed after staining with the *Bac*Light kit showing living (green fluorescence) and dead (red fluorescence) bacteria.

We investigated *in vitro* biofilm formation by 11A IPSD isolates with the pneumococcal R6 strain included as reference control. The biofilm forming capacity of invasive clinical isolates of serotype 11A was lower than that of the R6 strain probably due to differences in capsule expression, as the R6 strain is a non-encapsulated strain (Fig 5). Isolates of ST6521^11A^ showed a higher ability to produce biofilms than ST838^11A^ or ST62^11A^ (Fig 5).

## Discussion

Although pneumococcal conjugate vaccines are a proven success against targeted serotypes, considerable disease is also caused by certain non-vaccine serotypes which showed increased disease association in recent years [1,31–34]. One example was the emergence of the multi-drug resistant clone CC320 expressing serotype 19A, which was disseminated worldwide shortly after PCV-7 implementation, causing IPD in children and adults [5]. Our study shows some increased proportion of IPD caused by serotype 11A pneumococcal strains, especially in adult patients. The timing of this increased proportion coincides in time with the introduction of broader conjugate vaccines in Spain (PCV-10 and PCV-13). The proportion of serotype 11A associated with non-invasive pneumococcal disease has also increased in Spain during the period 2000–2012 (from 3.3% to 9.3% for adults (*P* <0.001) and from 0% to 10.8% in children (*P* <0.01) (data not shown). It is very important to closely monitor serotypes such as 11A that are already associated with a significant proportion of post-PCV pneumococcal disease and have unknown future potentials to cause greater disease burdens. Molecular characterization revealed the existence of three different genotypes, the well-known β-lactam susceptible clone ST62^11A^, and two PEN- and AMX-resistant emerging clones, ST838^11A^ and ST6521^11A^. Both β-lactam-resistant genotypes are variants of Spain^9V^-ST156 clone, which has been one of the most successful pneumococcal clones disseminated worldwide before PCV-7 introduction [5,32,35–37]. Serotype 11A variants of the ST156 clonal complex clone were described previously in Israel [32]. Since the 1980s in Spain, strains of this clonal complex have typically been associated with serotypes 9V and 14 (both included in current PCVs). Among adults, these strains caused the highest proportion (11.6%) of IPD [6]. The emergence of penicillin-non-susceptible serotype 11A strains of the highly successful ST156 lineage is of concern. These strains are not targeted by vaccines, and the majority of these isolates were AMX-resistant following non-meningitis criteria, which could preclude the use of this antibiotic in the treatment of pneumococcal disease in the community. The ST838 genotype associated with AMX-resistance was first described among serotype 9V isolates in the late 1990s [33,38], and is the putative ancestor of the 11A-ST838 variant. Although the ST6521 and ST838 variants differ in MLST genotype at only one locus, their different behaviors in reaction to the human immune response suggest additional genomic differences [8]. Our results suggest that the ST838^11A^ served as an intermediate strain that may have accumulated additional selectively advantageous changes during its conversion to ST6521^11A^.

Innate and adaptive immunity are critical factors for host defense during IPD, and clearance of pneumococci from the bloodstream is greatly dependent on opsonization by complement components and phagocytosis [10,11]. Clinical isolates of the ST62^11A^ clone showed an increased ability to avoid CP activation relative to the resistant 11A strains described in this study by targeting C1q and C4BP, leading to an impaired recognition by the key complement component C3b. This is of great relevance because the CP is the dominant pathway in complement activation and phagocytosis of *S*. *pneumoniae* [13,14]. Of the two PEN-resistant 11A clones, ST6521^11A^ avoided the immune response more efficiently than isolates of ST838^11A^. Although the numbers of isolates investigated are small, the observed difference in interaction with the CP might explain the increased incidence of ST6521^11A^ in IPD relative to ST838^11A^. This is important because a clear correlation exists between resistance to phagocytosis and carriage, where isolates more resistant to neutrophil clearance have an advantage to persist in the nasopharynx [7].

Antibiotic resistance is likely to provide a selective advantage in the nasopharyngeal reservoir, assuming that this advantage outweighs imposed fitness cost [39]. Biofilm formation provides an advantage during nasopharyngeal colonization or during the early steps of cellular attachment for dissemination, providing a mechanism for the avoidance of the immune response and reducing exposure to antibiotics [15,40]. The ability of pneumococcal serotypes to form biofilm could be used to predict the expansion of non-vaccine serotypes [16,41]. Invasive strain of ST6521^11A^ showed a higher capacity to form biofilms than strains of ST838^11A^ and ST62^11A^, which has possibly contributed to the emergence of this clone in the last few years. It is also possible that variation in capsule expression lead to some of these differences. For example, differences in capsule expression at different stages of biofilm formation could affect this process. It should be noted that under the conditions used for serotyping, the strains appeared qualitatively similar in expression of capsule. There is considerable accumulated data that indicates that features besides CPS expression modulate biofilm formation and interactions with complement-mediated immunity [16,29,41,42].

Continued strain surveillance studies and characterization of emergent disease-causing strains is necessary in order to gain a better understanding of what features lead to the success of individual successful pneumococcal clones.
